# Prescriber’s Preferences for Digital Health Applications in Mental Health Care: Cross-Sectional Best-Worst Scaling Study of General Practitioners and Psychotherapists in Germany

**DOI:** 10.2196/99203

**Published:** 2026-07-08

**Authors:** Carsten Volland, Felix Plescher, Pascal Raszke, Luisa Friedrich, Christian Speckemeier, Sarah Schlierenkamp, Michael Minor, Carina Abels, Klemens Höfer, Jürgen Wasem, Josepha Katzmann, Udo Schneider, Anja Wadeck, Sophia Zander, Anna Bußmann

**Affiliations:** 1 Essener Forschungsinstitut für Medizinmanagement (EsFoMed) GmbH Essen, North Rhine-Westphalia Germany; 2 Institute for Health Care Management and Research University of Duisburg-Essen Essen, North Rhine-Westphalia Germany; 3 German Psychotherapists Association (DPtV) Berlin, Berlin Germany; 4 Techniker Krankenkasse (Germany) Hamburg, Hamburg Germany

**Keywords:** digital health, mental health services, primary health care, psychotherapy, decision-making, health services research, mobile apps, Germany, choice behavior, surveys, questionnaires

## Abstract

**Background:**

Mental disorders affect nearly one-third of adults in Germany, with a 12-month prevalence of approximately 28%. Following Germany’s 2019 Digital Care Act, digital health applications (Digitale Gesundheitsanwendungen [DiGA]) became reimbursable interventions by the statutory health insurance for mental health conditions. However, adoption remains uneven. General practitioners (GPs) issue most mental health DiGA prescriptions, while psychotherapists or psychiatrists prescribe far fewer, even though most DiGA target mental health. Existing studies imply profession-specific barriers but lack quantitative evidence on preference drivers or remain descriptive. Whether and how these preferences differ across professional groups has not been systematically quantified.

**Objective:**

This study aimed to quantify and compare GPs’ and psychotherapists’ or psychiatrists’ preferences for factors associated with DiGA prescription decisions using best-worst scaling (BWS).

**Methods:**

A cross-sectional BWS study was conducted among outpatient GPs and psychotherapists or psychiatrists. Eleven DiGA objects were evaluated using a balanced incomplete block design. Preferences were analyzed using conditional logit regression. Likelihood ratio tests assessed differences between professional groups, with stratified models estimating group-specific odds ratios (OR) and 95% CIs.

**Results:**

Of 484 respondents (244 GPs and 240 psychotherapists or psychiatrists), 408 completed the BWS experiment. Group-specific ORs ranged from 0.47 to 1.72 (likelihood ratio test: *χ*^2^_10_=328.76; *P*<.001), suggesting that GPs and psychotherapists or psychiatrists operate within a broadly shared evaluative space but assign different relative emphasis to specific factors influencing DiGA prescription decisions. Relative to the reference object (intuitive usability for patients), GPs most strongly preferred scientific recommendations (OR 1.72, 95% CI 1.42-2.09) and patients’ interest in DiGA (OR 1.70, 95% CI 1.40-2.06) while significantly showing lower preferences for cross-device availability (OR 0.57, 95% CI 0.47-0.69) and access to patient-entered data (OR 0.47, 95% CI 0.39-0.57). Psychotherapists or psychiatrists showed different preference patterns, most strongly preferring device availability (OR 1.47, 95% CI 1.22-1.76) and contact points for technical support (OR 1.36, 95% CI 1.13-1.64) while showing significantly lower preference for alignment with scientific recommendations (OR 0.75, 95% CI 0.62-0.90).

**Conclusions:**

Professional role is a source of preference heterogeneity for factors associated with DiGA prescription decisions. GPs assigned comparatively greater weight to evidence-based recommendations and patient interest, while psychotherapists or psychiatrists emphasized technical integration feasibility and peer experience. These structured differences in priority gradients indicate that uniform implementation approaches may not adequately reflect the evaluative frameworks of both professional groups.

## Introduction

Mental disorders represent a growing public health burden in Germany, with a 12-month prevalence of approximately 28% among adults [1]. Following the 2019 Digital Care Act (Digitale-Versorgung-Gesetz), digital health applications (Digitale Gesundheitsanwendungen [DiGA]) have emerged as a novel tool intended to support the monitoring or treatment of a wide range of conditions [2]. To be listed in the official DiGA directory and eligible for reimbursement by the statutory health insurance (SHI), applications must demonstrate a positive health care effect (“positiver Versorgungseffekt”), which is evaluated by the Federal Institute for Drugs and Medical Devices (Bundesinstitut für Arzneimittel und Medizinprodukte). Despite this supportive regulatory framework and almost 6 years of availability, adoption remains uneven, particularly across professional prescriber groups [3]. The growing availability of digital mental health tools, including smartphone-based interventions, guided self-help programs, or artificial intelligence–assisted applications, has generated considerable interest alongside ongoing debates about clinical effectiveness, implementation barriers, and professional roles in digitally augmented care [4]. Within this broader landscape, DiGA occupy a distinctive position as the first formally reimbursable and evidence-evaluated digital health intervention category in Germany.

In principle, patients can access DiGA through either a prescription by a physician, psychotherapist, or via direct application to their statutory health insurance fund (SHIF). In practice, DiGA access is predominantly mediated by health care professionals, and prescribing patterns differ noticeably by professional group. Data from Germany’s largest SHIF (Techniker Krankenkasse) indicate that 37.6% of all DiGA are prescribed by general practitioners (GPs), whereas only 14.7% are prescribed by psychotherapists or psychiatrists [5]. Aggregated data from all SHIFs further demonstrate that this imbalance is even more pronounced in the mental health domain, where the GP-to-psychotherapist or psychiatrist prescribing ratio approximately doubles. Nearly 50% of all DiGA prescriptions are issued by GPs, while only around 10% are prescribed by psychotherapists or psychiatrists, and less than 10% are accessed directly by patients via their SHI [3]. This imbalance is also reflected in prescribing patterns for individual mental health DiGA. Deprexis and Somnio, for example, are 2 of the most frequently prescribed mental health DiGAs and are predominantly issued by GPs (55% and 46% of prescriptions, respectively), while psychotherapists and psychiatrists combined account for only 31% and 25%, respectively [3]. Notably, in 2024, approximately 55,000 GPs and 41,000 contracted psychotherapists participated in outpatient care in Germany [6], suggesting that the prescribing gap cannot be explained by differences in workforce size alone.

This distribution is striking given that mental disorders represent the largest therapeutic domain among the listed DiGA. As of April 2026, about half of all approved DiGA applications (30 of 59) target mental or behavioral disorders, including depression, anxiety, and stress-related syndromes [7]. The mismatch between the clinical focus of available DiGA and the professional groups that most actively prescribe them raises important questions about barriers to adoption and differential integration into routine care.

The average waiting time in Germany between initial contact with a psychotherapist and the start of long-term psychotherapeutic treatment is approximately 18 weeks [8]. During this period, patients may experience substantial symptom burden [9]. DiGA could help bridge this gap by providing low-threshold support. However, low prescribing rates across professional groups may contribute to limited use of digital interventions in the care pathways, as patients rarely access DiGA by themselves.

GPs, among other physicians, and psychotherapists or psychiatrists both hold prescribing authority for DiGA targeting mental health conditions and thus function as gatekeepers to DiGA access for patients with mental disorders, yet their roles and treatment contexts differ substantially. In Germany’s typical mental health care pathway, GPs often serve as the first point of contact, conducting initial assessments during brief consultations, often alongside somatic care, before referring patients to psychotherapists or psychiatrists for longer, structured psychotherapeutic treatment [10-13]. These differences in workflow, depth of patient interaction, and therapeutic responsibility may be relevant for understanding variation in how digital health applications are perceived.

Empirical evidence from Germany suggests that both professional groups express cautious interest in DiGA alongside specific concerns. GPs generally hold carefully positive attitudes toward DiGA [14]. Qualitative and quantitative studies by Wangler and Jansky [11,15] found that GPs view DiGA as reliable tools that may enhance patient compliance. However, GPs reported insufficient information about available applications, unclear certification processes, and challenges integrating DiGA into primary care workflows. Despite these reported challenges, recent survey studies from 2023 to 2024 showed that 59.4% of surveyed family doctors [16] and 31% of internal medicine practitioners [17] had already prescribed a DiGA.

Evidence from psychotherapists suggests that their evaluation of DiGA is shaped by the specific demands of psychotherapeutic care, including the particular needs of patients with mental health conditions. Qualitative research suggests that psychotherapists or psychiatrists regard internet- and mobile-based interventions more as complementary to traditional face-to-face psychotherapy rather than as integral components of routine care. While they acknowledge potential benefits, they also raise concerns about limited individualization, insufficient therapeutic depth, and technical or usability-related barriers that may disrupt ongoing therapeutic relationships [18]. Complementing these qualitative insights, evidence from a recent survey study indicates that prescribing behavior among psychotherapists or psychiatrists is systematically shaped by professional and structural factors, with substantially lower prescribing odds among psychodynamic and systemic therapists compared to those with a behavioral orientation [19], which may be related to the fact that most approved mental health DiGA are primarily based on cognitive behavioral therapy principles [20]. However, around 30% of the respondents reported that they prescribe DiGA [19].

Taken together, the recent evidence indicates that differences in DiGA acceptance in Germany are likely shaped by profession-specific expectations and concerns. However, most prior research has predominantly relied on qualitative or exploratory approaches that focus on general attitudes toward DiGA rather than on the systematic quantification of object-level trade-offs [14,15,18] or has not compared GPs to psychotherapists or psychiatrists [19]. As a result, robust quantitative evidence on which specific DiGA objects most strongly influence prescribing decisions and how these priorities differ between professional groups remains limited. The aim of this study is to address this gap by systematically eliciting and comparing the preferences of GPs and psychotherapists or psychiatrists regarding DiGA for mental disorders. Using a best-worst scaling (BWS) approach, we quantify the relative importance of key DiGA objects and identify profession-specific preference patterns to help create informative strategies that enable patients to benefit from digital mental health interventions.

## Methods

### Study Design

The BWS experiment was embedded in a cross-sectional mixed-mode survey conducted among GPs and psychotherapists or psychiatrists with SHI authorization working in outpatient care. The survey was part of the research project “Implementation of the potential of digital health applications in outpatient care for mental disorders (DiGAPsy)” funded by the federal joint committee (Funding code: 01VSF22029) [21].

### Data Collection and Procedures

A random sample of 2000 individuals for each group was drawn using a commercial address provider. All contacted GPs and psychotherapists or psychiatrists received a cover letter with detailed information on the study and data protection, along with a paper questionnaire and a prepaid return envelope. The letter also included a web link and QR code for online participation. Due to low participation rates in the GPs, incentivized surveys were sent out to 2 additional samples of 500 GPs drawn from the same commercial address provider and contacted using the same procedure. To further increase participation, the survey was additionally distributed through professional networks. Data collection took place from April 2024 to April 2025. Of 3000 contacted GPs, 244 participated (24.2% online, 75.8% paper). Of 2000 contacted psychotherapists or psychiatrists, 240 participated (58.8% online, 41.2% paper). Because the survey was additionally distributed through professional networks, precise response rates cannot be determined. However, based on the initial random samples alone (244/3000 GPs; 240/2000 psychotherapists or psychiatrists), maximum response rates were approximately 8% and 12%, respectively.

### Ethical Considerations

Participation was voluntary and anonymous. Informed consent was implied by the completion and return of the questionnaire, following the provision of detailed information about the study and data protection. Ethical approval was granted by the Ethics Committee of the Medical Faculty of the University of Duisburg-Essen (23-11209-BO). All procedures complied with data protection regulations and the Declaration of Helsinki [22]. Participants received no compensation for participation, with the exception of GP participants recruited through the additional random samples and through professional networks, who received a €20 (US $22.14; Date 01.10.2024) voucher as compensation to improve response rates, which had been low in the initial GP sample, approved by the Ethics Committee.

### Object Development

The object set was developed within the DiGAPsy project based on a scoping review identifying barriers to digital or mobile health integration into outpatient mental health care; focus groups with GPs, psychotherapists, and patients exploring perceived barriers and facilitators to DiGA use; and expert interviews with representatives from patient organizations, health care providers, statutory health insurers, and DiGA manufacturers examining implementation challenges and access pathways [21,23]. Following iterative team discussions, 11 objects were identified representing key conditions for DiGA prescription and use in outpatient mental health care. These objects covered technical, clinical, workflow-related, and patient-related aspects (see Table 1). The 11 objects represent a range of factors relevant to DiGA prescription decisions, encompassing both intrinsic application characteristics such as technical reliability and usability and external contextual factors such as patient interest and prior reputation. The development process was guided by established recommendations for qualitative object development in stated preference research [24]. After object development, the survey and the BWS component underwent cognitive pretesting to ensure the (construct) validity of the items and objects. The pretesting followed a 2-stage think-aloud protocol (n=7; March/April 2024), in which participants were asked to verbalize their thought processes while completing the questionnaire. Feedback was used to refine item wording and survey structure.

**Table 1:** Best-worst scaling (BWS) objects.

Number	Object
1	Having continuous access to patient-entered data (with consent).
2	Availability of the DiGA^a^ on different devices.
3	Reimbursement of DiGA-related work.
4	Availability of a contact point for technical or content-related questions.
5	Alignment of DiGA content with current scientific recommendations.
6	Ability to tailor DiGA content to individual patient needs.
7	Technical reliability of the DiGA.
8	Patient interest in using the DiGA.
9	Intuitive usability for patients.
10	Permanent (not provisional) listing of the DiGA in the DiGA directory.
11	Positive prior information or reputation regarding the DiGA.

^a^DiGA: Digitale Gesundheitsanwendungen.

### Experimental Design

A BWS Case 1 design was chosen to observe the relative importance of factors influencing GPs’ and psychotherapists’ or psychiatrists’ decisions to prescribe DiGA for mental health conditions. Case 1 BWS is suitable when the aim is to assess the relative value of a predefined set of standalone objects rather than object profiles or combinations [25,26]. Thus, BWS is a standard approach for early-stage preference elicitation in health research, as it requires respondents to make forced choices regarding their priority preference, and it yields interval-scaled, preference-weighted estimates that are more discriminating than classic rating-based approaches [27,28].

A balanced incomplete block design was applied, generated using SAS V9.4 (SAS Institute Inc). The design comprised 11 choice sets, each of which contained 5 of the 11 objects. Design parameters were optimized to ensure statistical efficiency. Each object appeared exactly 5 times across all choice sets, with an object frequency of r=5. Each pair of objects appeared together exactly twice, having a pairwise frequency of λ=2. In total, the design achieved 100% block D-efficiency and 88% treatment D-efficiency, which indicates a high precision for parameter estimation.

To reduce respondent burden, the 11 choice sets were divided into 2 survey versions (Group A and Group B) using a balanced blocking strategy. Group A received 6, and Group B received 5 choice sets, resulting in a total of 4 different versions (2 per professional group). Respondents were randomly assigned to one of the 2 versions, while randomization was performed separately for GPs and psychotherapists or psychiatrists to ensure balanced representation across both professional groups. With this blocking approach, a minor imbalance in the number of sets per respondent was created. However, the overall design maintained adequate object frequency (r=5), consistent pairwise co-occurrence (λ=2), and high statistical efficiency, achieving 100% block D-efficiency and 88% treatment D-efficiency, which meets methodological standards for object-case BWS designs when analyzed with conditional logit models that explicitly condition on the choice set [25]. The 2 survey versions differed only in choice set composition but contained the same 11 objects. Data from both versions were pooled for analysis, as the conditional logit approach accounts for the specific choice sets presented to each respondent.

Sample size was determined based on the overall DiGAPsy study protocol, which specified a minimum of 201 participants per professional group to detect medium effect sizes with 90% power and an α of .05 [21]. No separate power calculation was conducted for the BWS component. The achieved samples of 206 GPs and 202 psychotherapists or psychiatrists meet this prespecified threshold.

### Statistical Analysis

Best-worst (BW) responses were analyzed using conditional logit regression (*clogit* function from the survival package in R; R Foundation for Statistical Computing) [29], the standard analytical approach for Case 1 BWS that provides interpretable estimates of the relative importance of each object on a common latent scale [26,30]. This approach conditions on the offered choice set and leverages within-set comparisons only, making it robust to the minor imbalance in the number of choice sets between survey versions. The resulting parameters indicate the strength and direction of each object’s influence relative to a reference object or level, enabling comparisons across professional groups.

The model estimates utility parameters β for each object. Following the sequential BW coding, object indicators are entered as +1 for best-choice tasks and -1 for worst-choice tasks, yielding a single conditional logit:

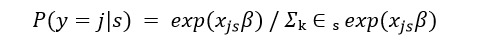



where *x*_js_ is the vector of object indicators for the object j in choice set s, with sign determined by the task type (+1 best and −1 worst). This ensures that a single set of β parameters captures the full preference scale from both best and worst responses. Following the sequential BW approach [30], best and worst responses were jointly analyzed within a single conditional logit model. For each choice set, the best and worst selections were restructured into a unified long-format dataset, with the attribute indicators for worst choices sign-reversed so that a single set of utility parameters captures the full preference scale. This sequential coding ensures that the best and worst responses contribute symmetrically to parameter estimation. “Intuitive usability for patients” was chosen as the reference level (β=0) because preliminary analysis showed it demonstrated moderate preference intensity in both professional groups, facilitating balanced interpretation of more and less preferred objects. All coefficients, therefore, represent log-odds relative to this reference. The *clogit* function is mathematically equivalent to a multinomial logit model when applied to BWS data structured as choice sets and yields identical parameter estimates. Exponentiated coefficients are reported as odds ratios (OR) with 95% CIs, representing relative preference weights. An OR >1 indicates that an object is more strongly preferred relative to the reference, while an OR <1 indicates weaker preference. For example, a coefficient of β=0.5 for “patient interest” indicates that this object is exponential (0.5)=1.65 times more likely to be chosen as “best” compared to the reference, that is, “intuitive usability for patients,” while holding the choice set constant.

Data from both survey versions (Group A and Group B) were pooled for analysis, as conditional logit explicitly accounts for the specific choice sets presented to each respondent. To verify that the blocking strategy did not systematically affect preferences, a base model (without survey version interactions) was compared to an interaction model (item × survey version) using a likelihood ratio test (LRT). The test showed no significant differences between versions (*χ*^2^_10_=4.60; *P*=.92), justifying the pooled analysis. The Akaike information criterion (AIC) also favored the simpler base model (AIC=13,266) over the interaction model (AIC=13,284). To examine differences in object preferences between GPs and psychotherapists or psychiatrists, an interaction model including all pairwise interactions between objects and professional role (item × role) was estimated. The significance of these interactions was also assessed using an LRT comparing the interaction model to the base model without role interactions.

Additionally, we computed aggregate best-worst (BW) scores using counting analysis as a descriptive supplement. The BW score for each object was calculated as the number of times chosen as “most important” minus the number of times chosen as “least important,” standardized across objects. Statistical significance was set at *P<*.05. All analyses were conducted using R (version 4.5.0) [31] with the *survival* [29], *support.BWS* [32], and *tidyverse* [33] packages.

To assess the robustness of the findings, the role-specific model was re-estimated under 2 alternative reference levels. To evaluate the assumption of preference homogeneity within professional groups, mixed logit models were estimated separately for GPs and psychotherapists or psychiatrists using 500 Halton draws and person-level random effects on all 10 item coefficients (R package *mlogit* [34]). For sensitivity analysis, the false discovery rate (FDR; Benjamini-Hochberg method) was used to correct for multiple testing [35,36]. In addition to uncorrected *P* values, Benjamini-Hochberg FDR-corrected *P* values are reported for the models as a conservative transparency measure and not as a correction for multiple testing, as all coefficients are estimated jointly within a single conditional logit model and therefore do not constitute a classical multiple testing problem.

## Results

### Participants’ Characteristics

Of the 244 GPs and 240 psychotherapists or psychiatrists who returned a questionnaire, a total of n=206 GPs and n=202 psychotherapists or psychiatrists completed the BWS component with no missing responses and were eligible for analysis. Most GPs were older than 50 (65.5%) years, whereas the majority of psychotherapists or psychiatrists were between 31 and 50 years old (54%). Psychotherapists or psychiatrists were predominantly female (75.2%) compared to GPs (50.7%) and were more likely to practice in large cities (52.9% vs 34.8%). Almost all GPs worked full-time (90.2%), whereas psychotherapists or psychiatrists were evenly split between full-time (49.0%) and part-time (50.5%). Most psychotherapists or psychiatrists were nonmedical psychotherapists (90.6%; see Table 2). The psychotherapist or psychiatrist sample was thus dominated by psychological psychotherapists. According to Kassenärztliche Bundesvereinigung (KBV) data for 2024, physician psychotherapists comprise approximately 14% of all contracted psychotherapists in Germany [6], suggesting a moderate underrepresentation in this sample (9.4%). Given the small subgroup size, separate analyses by psychotherapist or psychiatrist type were not feasible.

**Table 2:** Characteristics of general practitioners (GPs) and psychotherapists or psychiatrists.

Characteristics			GPs (n=206)		Psychotherapists or psychiatrists (n*=*202)
Age (years), n (%)					
	21-30	2 (1.0)		2 (1.0)	
	31-40	21 (10.3)		48 (23.8)	
	41-50	48 (23.5)		61 (30.2)	
	51-60	74 (36.3)		44 (21.8)	
	>60	59 (28.9)		47 (23.3)	
Sex, n (%)					
	Male	94 (45.9)		45 (22.3)	
	Female	104 (50.7)		152 (75.2)	
	No information	7 (3.4)		3 (1.5)	
	Other	0 (0.0)		2 (1.0)	
Location of practice, n (%)					
	Rural community (under 5000 residents)	36 (17.6)		9 (4.5)	
	Small town (5000 to <20,000 residents)	44 (21.6)		41 (20.3)	
	Medium-sized town (20,000 to <100,000 residents)	53 (26.0)		45 (22.3)	
	Large city (100,000 to <480,000 residents)	27 (13.2)		51 (25.2)	
	Large city (480,000 residents or more)	44 (21.6)		56 (27.7)	
Employment, n (%)					
	Full-time	184 (91.1)		99 (49.3)	
	Part-time	18 (8.9)		102 (50.7)	
Professional background, n (%)					
	Psychological psychotherapist	—^a^		183 (90.6)	
	Physician psychotherapist/psychiatrist	—		19 (9.4)	

^a^Not applicable.

### BWS Analysis

No significant differences were found between survey versions (LRT: *χ*^2^_10_=4.60; *P*=.92), and both groups showed similar preference patterns with all objects having effects in the same direction (see Multimedia Appendix 1). Descriptive BW scores confirmed that “Intuitive usability for patients” occupies a middle position in the preference distribution across both groups, supporting its selection as the reference level for the conditional logit models.

When examining the pooled results from the conditional logit model without interaction effects, this preference pattern was confirmed (see Table 3). However, the modeled approach revealed that only “Patient interest in using DiGA” (OR 1.361, 95% CI 1.193-1.553) and “Ability to tailor content to patient needs” (OR 1.204, 95% CI 1.057-1.372) were significantly preferred over “Intuitive usability for patients,” whereas only “Permanent listing in DiGA directory” (OR 0.855, 95% CI 0.75-0.975) and “Continuous access to patient-entered data” (OR 0.765, 95% CI 0.671-0.871) were significantly less preferred than the reference category. “Contact point for technical/content questions” (OR 1.145, 95% CI 1.005-1.304) showed no significant preference after FDR correction. The pooled model demonstrated acceptable fit (AIC=13,266.4).

**Table 3:** Results of the pooled best-worst scaling (BWS) analysis (conditional logit model; n=408).

Object	BW^a^ Score	Mean BWS^b^ (95% CI)	OR^c^ (95% CI)	*P* value^d^	*P*-FDR^e,f^
Patient interest in using DiGA^g^	125	0.306 (0.204 to 0.408)	1.361 (1.193-1.553)	<.001	<.001
Ability to tailor content to patient needs	76	0.186 (0.091 to 0.281)	1.204 (1.057-1.372)	.005	.02
Contact point for technical/content questions	53	0.13 (0.053 to 0.207)	1.145 (1.005-1.304)	.04	.08
Alignment with scientific recommendations	48	0.118 (0.006 to 0.23)	1.130 (0.992-1.289)	.07	.11
Technical reliability	38	0.093 (0.023 to 0.163)	1.107 (0.971-1.262)	.13	.16
Reimbursement of DiGA-related effort	–30	–0.074 (–0.17 to 0.022)	0.948 (0.831-1.081)	.42	.42
Availability on different devices	–42	–0.103 (–0.217 to 0.011)	0.924 (0.811-1.053)	.24	.27
Positive prior information/reputation	–59	–0.145 (–0.241 to –0.049)	0.888 (0.779-1.013)	.08	.11
Permanent listing in DiGA directory	–75	–0.184 (–0.279 to –0.089)	0.855 (0.75-0.975)	.02	.05
Continuous access to patient-entered data	–127	–0.311 (–0.419 to –0.203)	0.765 (0.671-0.871)	<.001	<.001
Intuitive usability for patients	–7	–0.017 (–0.107 to 0.073)	Reference level^h^	—^i^	—

^a^BW: best-worst.

^b^Mean BWS: average best-worst score per respondent. Model fit: Akaike information criterion 13,266.4; log-likelihood –6623.2.

^c^OR: odds ratio; >1 indicates stronger preference relative to reference and <1 indicates weaker preference.

^d^*P* value: uncorrected 2-sided *P* value.

^e^*P*-FDR: Benjamini-Hochberg–corrected *P* value across all 10 pooled tests.

^f^FDR: false discovery rate.

^g^DiGA: Digitale Gesundheitsanwendungen.

^h^Reference category is “Intuitive usability for patients.”

^i^Not applicable.

Table 4 shows the results of the conditional logit model stratified by professional role. Allowing object preferences to vary by professional role significantly improved model fit compared with the pooled model (LRT: *χ*^2^_10_=328.76; *P<*.001). The interaction model yielded a substantially lower AIC (12,957.7) compared with the pooled model (13,266.4), corresponding to a ΔAIC (difference in AIC) of −308.8, indicating strong evidence for preference heterogeneity between professional groups.

**Table 4:** Professional role-specific conditional logit model (n=408).

Object	GPs^a^ (n=206)		Psychotherapists or psychiatrists (n=202)	
	OR^b^ (95% CI)	*P*-FDR^c,d^	OR (95% CI)	*P*-FDR
Patient interest in using DiGA^e^	1.696 (1.399-2.057)	<.001	1.098 (0.91-1.324)	.41
Ability to tailor content to patient needs	1.343 (1.11-1.626)	.005	1.087 (0.905-1.306)	.44
Contact point for technical/content questions	0.955 (0.789-1.156)	.67	1.361 (1.132-1.636)	.003
Alignment with scientific recommendations	1.723 (1.424-2.086)	<.001	0.751 (0.623-0.904)	.005
Technical reliability	1.056 (0.872-1.278)	.64	1.158 (0.962-1.394)	.19
Reimbursement of DiGA-related effort	0.884 (0.729-1.071)	.30	0.999 (0.828-1.205)	.99
Availability on different devices	0.568 (0.469-0.689)	<.001	1.465 (1.217-1.764)	<.001
Positive prior information/reputation	0.63 (0.52-0.763)	<.001	1.219 (1.012-1.468)	.07
Permanent listing in DiGA directory	0.642 (0.53-0.778)	<.001	1.117 (0.928-1.344)	.33
Continuous access to patient-entered data	0.467 (0.385-0.567)	<.001	1.19 (0.99-1.43)	.11
Intuitive usability for patients	Reference level^f^	—^g^	Reference level	—

^a^GP: general practitioner.

^b^OR: odds ratio.

^c^*P*-FDR: false discovery rate–corrected *P* values. Benjamini-Hochberg–corrected *P* values applied across all 20 stratified tests (10 GP + 10 PSY) simultaneously. The role interaction model significantly improved the fit compared with the pooled model (likelihood ratio test: χ^2^_10_=328.8; *P*<.001). Model fit: Akaike information criterion (AIC) 12957.7; ΔAIC vs pooled –308.8.

^d^FDR: false discovery rate.

^e^DiGA: Digitale Gesundheitsanwendungen.

^f^Reference category is “Intuitive usability for patients.”

^g^Not applicable.

Among GPs, the strongest positive preferences were observed for alignment with scientific recommendations (OR 1.72, 95% CI 1.42-2.09), patient interest in using DiGA (OR 1.70, 95% CI 1.40-2.06), and the ability to tailor content to patient needs (OR 1.34, 95% CI 1.11-1.63). In contrast, GPs significantly dispreferred availability on different devices (OR 0.57, 95% CI 0.47-0.69), positive prior information or reputation (OR 0.63, 95% CI 0.52-0.76), permanent listing in the DiGA directory (OR 0.64, 95% CI 0.53-0.78), and continuous access to patient-entered data (OR 0.47, 95% CI 0.39-0.57).

Psychotherapists or psychiatrists showed a different preference pattern, significantly preferring “Availability on different devices” (OR 1.465, 95% CI 1.217-1.764), “Contact point for technical/content questions” (OR 1.361, 95% CI 1.132-1.636), and “Positive prior information/reputation” (OR 1.219, 95% CI 1.012-1.468). Most other objects showed preferences near neutral (OR≈1, CI includes 1), with “Alignment with scientific recommendations” being the only object significantly less preferred than the reference level (OR 0.751, 95% CI 0.623- 0.904).

**Figure 1:**
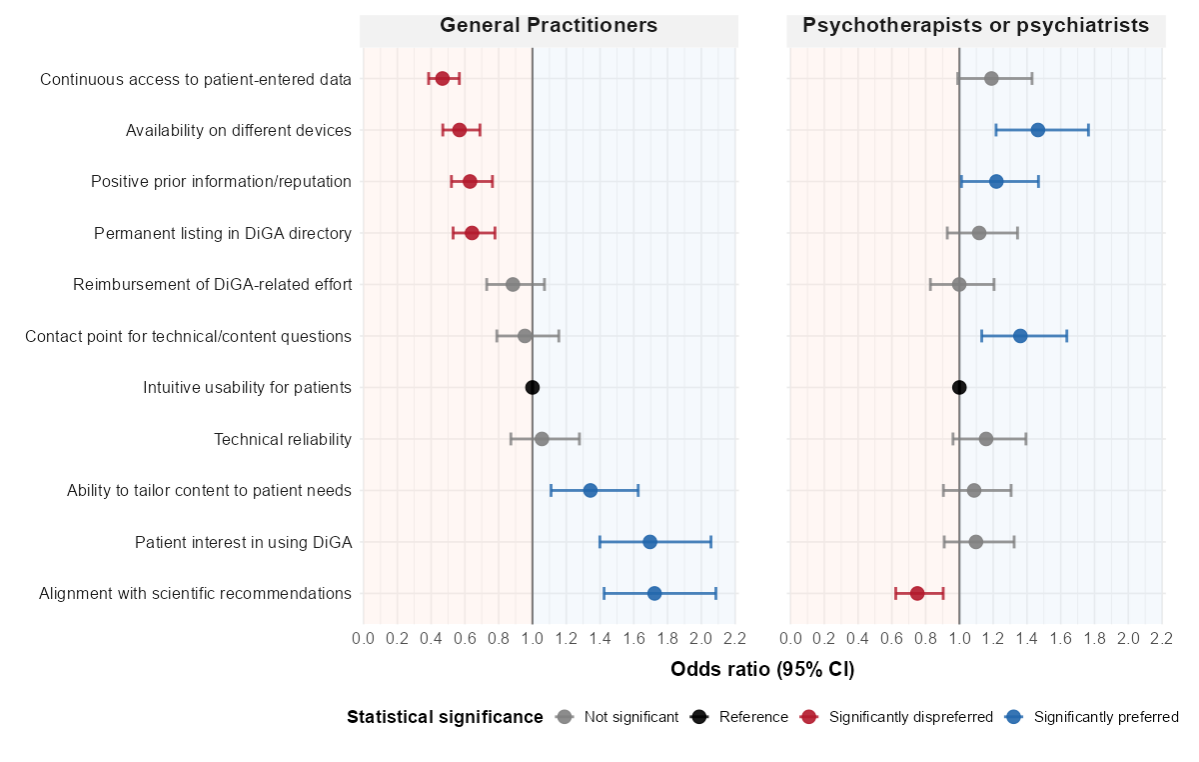
Group-specific odds ratios (ORs) with 95% CIs for DiGA objects by professional role. The reference category is “Intuitive usability for patients” (OR 1.0). Blue=significantly preferred over reference. Red=significantly dispreferred. Gray=not significant. Whiskers represent 95% CIs. DiGA: Digitale Gesundheitsanwendung (digital health application).

Figure 1 illustrates the preference rating of GPs and psychotherapists or psychiatrists. It becomes evident that GPs and psychotherapists or psychiatrists showed distinct preference patterns regarding DiGA. GPs strongly valued scientific recommendations, while psychotherapists or psychiatrists dispreferred them, and GPs strongly dispreferred availability on different devices, while psychotherapists or psychiatrists found it to be most important. Descriptively, objects that GPs rated above the reference tended to be rated at or below the reference by psychotherapists or psychiatrists, and vice versa. However, this pattern reflects differences in relative priority ordering rather than diametrically opposed evaluations, as several psychotherapist or psychiatrist estimates had confidence intervals crossing 1, indicating indifference rather than rejection.

As sociodemographic variables (age, sex, practice location, and employment) differed between GPs and psychotherapists or psychiatrists, sensitivity analysis was performed. Three-way interactions (object × role × demographic) were tested for age, sex, practice location, and employment status. Correction for multiple testing was applied using the FDR method. As shown in Multimedia Appendix 2, sensitivity analysis identified age in particular as a significant moderator for preferences (*χ*^2^_20_=88.8; *P-*FDR<.001). Practice location (*χ*^2^_20_=37.0; *P-*FDR=.02) and employment status (*χ*^2^_20_=35.7; *P-*FDR=.02) also showed modest moderation effects, while sex had no significant influence (*χ*^2^_20_=25.3; *P-*FDR=.19). However, despite these demographic moderation effects, professional role remained the dominant predictor of preference heterogeneity (main effect *χ*^2^_10_=328.8; *P*<.001).

The preference rank-ordering was invariant to the choice of reference level across all 3 specifications (Spearman ρ≥0.964 for GPs, ρ=1.000 for psychotherapists or psychiatrists; Multimedia Appendices 3 and 4). Mixed logit sensitivity analysis confirmed directional consistency of mean preference parameters with the primary conditional logit estimates for all 10 objects in GPs and 9 of 10 objects in PSYs; the single discrepancy concerned reimbursement among PSYs, where both models indicated practical indifference (*clogit* OR 1.00, 95% CI 0.83-1.20; mixed logit mean OR 1.14; Multimedia Appendices 5, 6, and 7). The AIC of the mixed logit was substantially lower compared to the conditional logit (ΔAIC=−2057), confirming the presence of individual-level preference heterogeneity within both professional groups, while mean preference parameters remained directionally consistent with the primary estimates.

## Discussion

### Principal Findings

DiGA adoption has increased since its introduction in 2020, with recent surveys indicating rising prescription rates among both GPs and psychotherapists [16,17,19]. Despite this upward trend, differences in prescribing patterns across professional groups remain observable.

This BWS study identified statistically significant differences in how factors associated with DiGA prescription decisions are weighted by GPs and psychotherapists or psychiatrists. These findings should be interpreted as differences in relative priority gradients rather than diametrically opposed evaluative frameworks. Although interaction effects were robust (*χ*^2^_10_=328.76; *P*<.001), most odds ratios ranged between approximately 0.5 and 1.7, indicating moderate differences in relative weighting rather than extreme divergence in priorities. Rather than reflecting fundamentally different evaluative frameworks, the results indicate that GPs and psychotherapists or psychiatrists operate within a broadly shared evaluative space but assign different relative emphasis to selected DiGA characteristics. For example, GPs assigned comparatively greater weight to alignment with scientific recommendations and patient interest, whereas psychotherapists or psychiatrists assigned comparatively greater weight to cross-device availability and access to technical support structures. These contrasts represent differences in priority gradients rather than mutually exclusive value systems.

GPs’ prioritization of scientific recommendations may reflect the structural conditions and the clinical decision-making context. Primary care consultations in Germany are less than 8 minutes on average [10], often requiring rapid assessment and management decisions. In such contexts, reliance on established evidence and formal recommendations may be consistent with a pragmatic decision heuristic, although this interpretation remains speculative as the BWS design captures stated preference weights rather than decision processes directly. This interpretation is consistent with prior research showing that GPs and physicians in general cite insufficient information on implementing digital tools and the absence of trusted, neutral information platforms as major barriers to DiGA adoption [11,17].

The comparatively high importance assigned to patient interest among GPs further suggests a preference for shared or patient-initiated decision-making. Rather than actively promoting DiGA, GPs may prescribe when patients express explicit interest, potentially as a pragmatic strategy to mitigate the risk of low uptake or poor adherence. Previous studies show that digital literacy as well as DiGA usage is related to a younger age and a higher socioeconomic status [37,38]. The concurrent preference for tailoring content to patient needs supports this interpretation.

In contrast to the GPs, the preference structure observed among psychotherapists or psychiatrists may be consistent with a different treatment context. Psychotherapists or psychiatrists usually operate within longer, structured therapeutic encounters (usually 50 minutes following the Guideline of the Federal Joint Committee on the Provision of Psychotherapy [39]) and assume primary responsibility for the long-term management of mental disorders. Accordingly, DiGA may be more likely evaluated based on their feasibility for integration into psychotherapy rather than on external scientific recommendations or patient-initiated demand. Notably, psychotherapists or psychiatrists did not reject evidence alignment in absolute terms. Rather, they ranked it below other attributes such as usability and technical support. The significant OR of 0.75 indicates lower relative priority compared to the reference category, not opposition to evidence-based practice per se.

The strong preference for objects related to technical feasibility, shown in the strong preferences toward cross-device availability and clearly defined technical support, suggests that psychotherapists or psychiatrists prioritize whether a DiGA can be used reliably between sessions and maintained without disrupting therapeutic continuity. This interpretation is consistent with findings showing that psychotherapists or psychiatrists regard limited technical infrastructure as a central barrier in e-mental health adoption [40]. In addition, prescribing DiGA may be perceived as a shift in professional roles, with psychotherapists anticipating increased responsibility for supporting patients in the use of digital applications, including responding to technical or usage-related questions, which may contribute to concerns about implementation.

One interpretive caveat applies to the object “availability of the DiGA on different devices,” which was intended to capture cross-device accessibility for patients. In the psychotherapeutic context, some psychotherapists or psychiatrists may have understood this more broadly as referring to simultaneous access by both patient and therapist across devices, which would conceptually overlap with “continuous access to patient-entered data.” If this interpretation was prevalent, the observed PSY preference would at least partly reflect a preference for therapeutic data integration rather than cross-device patient use. Nevertheless, it is important to note that cognitive pretesting did not identify this as a systematic issue, and no participant flagged a dual interpretation during the think-aloud procedure. However, this post hoc ambiguity cannot be fully excluded and should be considered when interpreting the psychotherapist’s or psychiatrist’s preference pattern for this object. Furthermore, psychotherapists or psychiatrists showed stronger preferences for positive prior information or reputation, suggesting that peer recommendations and practical implementation experiences may carry particular weight in their evaluation process. This aligns with findings by Braun et al [41], who demonstrate that the acceptance of e-mental health services among psychotherapists is shaped by social influence. Stalujanis et al [19] further demonstrate that operating in a group practice increases the likelihood of prescribing DiGA compared to solo practices, suggesting that peer knowledge exchange facilitates adoption. It should further be noted that the psychotherapist or psychiatrist sample was dominated by psychological psychotherapists (90.6%), with physician psychotherapists moderately underrepresented relative to the KBV 2024 population benchmark of approximately 14% [6]. The observed preference pattern may therefore primarily reflect the evaluative framework of psychological psychotherapists. Whether physician psychotherapists and psychiatrists show distinct preference structures warrants investigation in future studies with larger subgroup samples.

In summary, GPs may relate to efficiency-oriented considerations emphasizing external validation and patient demand, whereas psychotherapists or psychiatrists appear to prioritize low technical barriers and support structures compatible with longer therapeutic relationships. These distinctions in relative preference patterns may, at least partially, relate to broader differences in workflow structures and professional roles. However, the cross-sectional design of the study precludes causal attribution.

Sensitivity analyses indicated that age and selected practice characteristics modestly moderated professional differences, with age showing the strongest effect. However, professional role remained the dominant predictor of preference heterogeneity. These findings suggest that both professional context and generational factors may shape DiGA evaluation, although demographic moderation does not eliminate the observed role-based differences.

These findings suggest that differentiated information strategies tailored to professional context may warrant consideration, although the translation from stated preference weights to actionable implementation recommendations requires further empirical validation. For GPs, improving visibility of evidence-based recommendations and facilitating patient-facing information may be most effective, whereas for psychotherapists or psychiatrists, efforts should focus on perceived technical demands, ensuring reliable support structures, and leveraging peer networks for knowledge exchange. Future research should examine how these role-specific preferences translate into actual prescribing behavior (ie, revealed preferences) and whether targeted implementation strategies can improve uptake across both professional groups.

### Limitations

Several limitations should be considered when interpreting the findings of this study. First, the cross-sectional design prevents causal inference, and it cannot be determined whether the preferences cause the prescribing behavior or the prescribing experiences are shaping the preferences.

Second, given the nature of the BW experiment, preferences are measured hypothetically and are not real-world decisions or reported behavior; an intention-behavior gap is most likely to occur [42]. Future research should focus on comparing stated preferences with actual prescribing behavior (ie, revealed preferences).

Third, the sample combined random and convenience recruitment, raising concerns about a potential selection bias. Given that respondents self-selected into a survey explicitly focused on DiGA and that the survey was additionally distributed through professional networks, prescribers with a higher familiarity or even a more positive attitude toward digital health are more likely to have submitted the survey than the broader outpatient GPs and psychotherapists or psychiatrists in Germany. With maximum possible response rates between 8% and 12%, the sample is prone to nonresponse bias and therefore rather represents the relative priorities of digital-literate GPs or psychotherapists or psychiatrists than the full spectrum of prescribers’ preferences. To assess representativeness, sample demographics were compared to population-level data from the National Association of Statutory Health Insurance Physicians [6] (see Multimedia Appendix 8). Sex distributions closely matched the reference populations for both groups. Age distributions were broadly comparable, though both samples showed a moderate underrepresentation of professionals aged 60 years and older, suggesting that younger, potentially more digitally engaged professionals may have been somewhat more likely to participate. However, demographic comparisons with population benchmarks provide limited reassurance, as digital health attitudes are not adequately captured by age and sex distributions alone. Findings should therefore be interpreted as characterizing preference patterns among digitally engaged prescribers rather than as population-representative estimates.

Fourth, the reference category for the conditional logit models was selected through a data-driven approach rather than a priori theoretical specification. While choosing a middle-ranked object as a reference is standard practice in BWS analysis [25], the results remain sensitive to the reference level. However, robustness checks re-estimating the role-specific model under 2 alternative reference levels confirmed that the preference rank-ordering was fully invariant across all 3 specifications (Spearman ρ≥0.964 for GPs and ρ=1.000 for psychotherapists or psychiatrists, Multimedia Appendices 3 and 4), indicating that substantive conclusions are not sensitive to the choice of reference level. Finally, findings are specific to the German health care context, particularly the unique DiGA regulatory framework, and professional training structures. Aspects like GPs’ and psychotherapists’ or psychiatrists’ roles or consultation durations may differ substantially across countries. Generalizability to other digital health contexts or international settings remains uncertain and requires cross-national replication studies.

### Conclusion

This study demonstrates that professional role is a source of preference heterogeneity for factors associated with DiGA prescription decisions among German health care professionals. The different relative priorities, particularly regarding scientific evidence and device availability, suggest that uniform approaches to DiGA information and continuing education may not adequately address the needs of both professional groups. Targeted measures could include integrating DiGA evidence summaries into GP-oriented guideline platforms and providing technical support resources through psychotherapist professional associations. However, effective translation of these findings requires clearly identified actors—such as statutory health insurers, professional associations, or DiGA manufacturers—to develop and deliver group-specific information strategies.

## Data Availability

The data underlying this study cannot be made publicly available. The survey was conducted anonymously. Reidentification risks preclude data sharing in accordance with applicable data protection regulations. Aggregated results are presented in the manuscript. Requests for further information may be directed to the corresponding author.

## Appendix

Multimedia Appendix 1 Aggregate best-worst scores by survey group.

Multimedia Appendix 2 Sensitivity analysis: demographic moderation of professional role effects.

Multimedia Appendix 3 Robustness check: alternative reference categories—general practitioners.

Multimedia Appendix 4 Robustness check: alternative reference categories—psychotherapists.

Multimedia Appendix 5 Model fit comparison (Akaike information criterion).

Multimedia Appendix 6 Mixed logit sensitivity analysis—general practitioners.

Multimedia Appendix 7 Mixed logit sensitivity analysis—psychotherapists.

Multimedia Appendix 8 Sample representativeness.

## Abbreviations

AIC: Akaike Information Criterion
BW: best-worst
BWS: best-worst scaling
DiGA: Digitale Gesundheitsanwendung (digital health application)
FDR: false discovery rate
GP: general practitioner
KBV: Kassenärztliche Bundesvereinigung
LRT: likelihood ratio test
OR: odds ratio
SHI: statutory health insurance
SHIF: statutory health insurance fund
