# Supplementary material for: Prescriber’s Preferences for Digital Health Applications in Mental Health Care: Cross-Sectional Best-Worst Scaling Study of General Practitioners and Psychotherapists in Germany
Source: J Med Internet Res. 2026 Jul 8;28:e99203. doi: 10.2196/99203 (PMC13392533; doi:10.2196/99203)
Supplement: Multimedia Appendix 1 [file jmir_v28i1e99203_app1.doc]

Supplement 1: Aggregate best-worst scores by survey group.

| **Objects** | **Group A (6 sets)** | | | |  | **Group B (5 sets)** | | | |
| --- | --- | --- | --- | --- | --- | --- | --- | --- | --- |
|  | **Best** | **Worst** | **BW** | **Std BW** |  | **Best** | **Worst** | **BW** | **Std BW** |
| Patient interest in using DiGA | 219 | 113 | 106 | 0.137 |  | 69 | 50 | 19 | 0.089 |
| Ability to tailor content to patient needs | 99 | 61 | 38 | 0.098 |  | 133 | 95 | 38 | 0.059 |
| Contact point for technical/content questions | 56 | 35 | 21 | 0.054 |  | 98 | 66 | 32 | 0.050 |
| Alignment with scientific recommendations | 158 | 144 | 14 | 0.024 |  | 140 | 106 | 34 | 0.079 |
| Technical reliability | 50 | 51 | -1 | -0.002 |  | 76 | 37 | 39 | 0.091 |
| Intuitive usability for patients | 33 | 43 | -10 | -0.052 |  | 138 | 135 | 3 | 0.004 |
| Reimbursement of DiGA-related effort | 136 | 157 | -21 | -0.027 |  | 47 | 56 | -9 | -0.042 |
| Availability on different devices | 156 | 172 | -16 | -0.027 |  | 105 | 131 | -26 | -0.061 |
| Positive prior information/reputation | 87 | 117 | -30 | -0.052 |  | 85 | 114 | -29 | -0.068 |
| Permanent listing in DiGA directory | 89 | 144 | -55 | -0.095 |  | 67 | 87 | -20 | -0.047 |
| Continuous access to patient-entered data | 79 | 125 | -46 | -0.119 |  | 112 | 193 | -81 | -0.126 |

Std BW = Standardized Best-Worst score. No significant differences between survey version groups (LRT: χ²=4.60, *P*=.916).
