# Supplementary material for: Prescriber’s Preferences for Digital Health Applications in Mental Health Care: Cross-Sectional Best-Worst Scaling Study of General Practitioners and Psychotherapists in Germany
Source: J Med Internet Res. 2026 Jul 8;28:e99203. doi: 10.2196/99203 (PMC13392533; doi:10.2196/99203)
Supplement: Multimedia Appendix 2 [file jmir_v28i1e99203_app2.doc]

Supplement 1: Sensitivity analysis: Demographic moderation of professional role effects.

| **Demographic Moderator** | **χ²** | **df** | ***P* (uncorrected)** | ***P-FDR*** |
| --- | --- | --- | --- | --- |
| Age (≥50 vs <50) | 88.78 | 20 | <.001 | <.001 |
| Sex (female vs. male) | 25.34 | 20 | .189 | .19 |
| Practice location (urban vs rural) | 37.03 | 20 | .012 | .02 |
| Employment status (full-time vs. part-time) | 35.66 | 20 | .017 | .02 |

Note: Three-way interaction models (object × role × demographic) tested whether demographic characteristics modified professional group differences in DiGA object preferences. P-FDR: False discovery rate-corrected p-values (Benjamini-Hochberg method) for 4 tests.
