# Supplementary material for: Prescriber’s Preferences for Digital Health Applications in Mental Health Care: Cross-Sectional Best-Worst Scaling Study of General Practitioners and Psychotherapists in Germany
Source: J Med Internet Res. 2026 Jul 8;28:e99203. doi: 10.2196/99203 (PMC13392533; doi:10.2196/99203)
Supplement: Multimedia Appendix 3 [file jmir_v28i1e99203_app3.doc]

Supplement 1: Robustness Check: Alternative Reference Categories – General Practitioners.

|  | Ref: Intuitive usability |  | Ref: Technical reliability |  | Ref: Reimbursement |  |
| --- | --- | --- | --- | --- | --- | --- |
| Object | OR (95% CI) | Rank | OR (95% CI) | Rank | OR (95% CI) | Rank |
| Alignment with scientific recommendations | 1.72 (1.42–2.09)*** | 1 | 1.63 (1.35–1.98)*** | 1 | 1.95 (1.61–2.37)*** | 1 |
| Patient interest in using DiGA | 1.70 (1.40–2.06)*** | 2 | 1.61 (1.33–1.95)*** | 2 | 1.92 (1.58–2.33)*** | 2 |
| Ability to tailor content to patient needs | 1.34 (1.11–1.63)** | 3 | 1.27 (1.05–1.54)* | 3 | 1.52 (1.26–1.84)*** | 3 |
| Technical reliability | 1.06 (0.87–1.28) | 4 |  |  | 1.19 (0.99–1.45) | 4 |
|  | Reference | 5 | Reference | 4 | Reference | 7 |
| Contact point for technical/content questions | 0.96 (0.79–1.16) | 6 | 0.90 (0.75–1.10) | 6 | 1.08 (0.89–1.31) | 6 |
| Reimbursement of DiGA-related effort | 0.88 (0.73–1.07) | 7 | 0.84 (0.69–1.02) | 7 |  |  |
| Permanent listing in DiGA directory | 0.64 (0.53–0.78)*** | 8 | 0.61 (0.50–0.74)*** | 8 | 0.73 (0.60–0.88)** | 8 |
| Positive prior information/reputation | 0.63 (0.52–0.76)*** | 9 | 0.60 (0.49–0.72)*** | 9 | 0.71 (0.59–0.86)*** | 9 |
| Availability on different devices | 0.57 (0.47–0.69)*** | 10 | 0.54 (0.44–0.65)*** | 10 | 0.64 (0.53–0.78)*** | 10 |
| Continuous access to patient-entered data | 0.47 (0.39–0.57)*** | 11 | 0.44 (0.36–0.54)*** | 11 | 0.53 (0.44–0.64)*** | 11 |
| Intuitive usability for patients |  |  | 0.95 (0.78–1.15) | 5 | 1.13 (0.93–1.37) | 5 |

Note: OR = Odds Ratio; CI = Confidence Interval. *P<0.05, **P<0.01, ***P<0.001 (uncorrected). Spearman rank correlations: “Ref: Intuitive usability” vs “Ref: Technical reliability”: ρ=1.000; “Ref: Intuitive usability” vs “Ref: Reimbursement”: ρ=0.988; “Ref: Technical reliability” vs “Ref: Reimbursement”: ρ=0.964. High correlations indicate stable preference rank-ordering across reference specifications.
