# Supplementary material for: Prescriber’s Preferences for Digital Health Applications in Mental Health Care: Cross-Sectional Best-Worst Scaling Study of General Practitioners and Psychotherapists in Germany
Source: J Med Internet Res. 2026 Jul 8;28:e99203. doi: 10.2196/99203 (PMC13392533; doi:10.2196/99203)
Supplement: Multimedia Appendix 4 [file jmir_v28i1e99203_app4.doc]

Supplement 1: Robustness Check: Alternative Reference Categories – Psychotherapists.

|  | Ref: Intuitive usability |  | Ref: Technical reliability |  | Ref: Reimbursement |  |
| --- | --- | --- | --- | --- | --- | --- |
| Object | OR (95% CI) | Rank | OR (95% CI) | Rank | OR (95% CI) | Rank |
| Availability on different devices | 1.47 (1.22–1.76)*** | 1 | 1.27 (1.05–1.53)* | 1 | 1.47 (1.21–1.78)*** | 1 |
| Contact point for technical/content questions | 1.36 (1.13–1.64)** | 2 | 1.18 (0.97–1.42) | 2 | 1.36 (1.13–1.65)** | 2 |
| Positive prior information/reputation | 1.22 (1.01–1.47)* | 3 | 1.05 (0.87–1.27) | 3 | 1.22 (1.01–1.48)* | 3 |
| Continuous access to patient-entered data | 1.19 (0.99–1.43) | 4 | 1.03 (0.85–1.24) | 4 | 1.19 (0.99–1.44) | 4 |
| Technical reliability | 1.16 (0.96–1.39) | 5 |  |  | 1.16 (0.96–1.40) | 5 |
| Permanent listing in DiGA directory | 1.12 (0.93–1.34) | 6 | 0.96 (0.80–1.16) | 5 | 1.12 (0.92–1.35) | 6 |
| Patient interest in using DiGA | 1.10 (0.91–1.32) | 7 | 0.95 (0.78–1.15) | 6 | 1.10 (0.91–1.33) | 7 |
| Ability to tailor content to patient needs | 1.09 (0.90–1.31) | 8 | 0.94 (0.78–1.13) | 7 | 1.09 (0.90–1.31) | 8 |
| Reimbursement of DiGA-related effort | 1.00 (0.83–1.20) | 9 | 0.86 (0.71–1.04) | 9 |  |  |
| Alignment with scientific recommendations | 0.75 (0.62–0.90)** | 10 | 0.65 (0.54–0.78)*** | 10 | 0.75 (0.62–0.91)** | 10 |
| Intuitive usability for patients |  |  | 0.86 (0.72–1.04) | 8 | 1.00 (0.83–1.21) | 9 |

Note: OR = Odds Ratio; CI = Confidence Interval. *P<0.05, **P<0.01, ***P<0.001 (uncorrected). Spearman rank correlations: “Ref: Intuitive usability” vs “Ref: Technical reliability”: ρ=1.000; “Ref: Intuitive usability” vs “Ref: Reimbursement”: ρ=1.000; “Ref: Technical reliability” vs “Ref: Reimbursement”: ρ=1.000. High correlations indicate stable preference rank-ordering across reference specifications.
