# Supplementary material for: Prescriber’s Preferences for Digital Health Applications in Mental Health Care: Cross-Sectional Best-Worst Scaling Study of General Practitioners and Psychotherapists in Germany
Source: J Med Internet Res. 2026 Jul 8;28:e99203. doi: 10.2196/99203 (PMC13392533; doi:10.2196/99203)
Supplement: Multimedia Appendix 5 [file jmir_v28i1e99203_app5.doc]

Mixed logit models were estimated separately for GPs and PSYs using 500 Halton draws and person-level random effects on all ten item coefficients (R package mlogit). The substantially lower AIC of the mixed logit compared to the conditional logit (pooled CLogit AIC=13,266; pooled MXL AIC=11,210; GP MXL AIC=5,531; PSY MXL AIC=5,498) confirms the presence of individual-level preference heterogeneity within both professional groups.

Supplement 1: Model Fit Comparison (AIC)

| Model | AIC |
| --- | --- |
| Conditional Logit (pooled) | 13266.4 |
| Mixed Logit (pooled) | 11209.6 |
| Mixed Logit (GPs) | 5530.7 |
| Mixed Logit (PSYs) | 5498.2 |

Note: Lower AIC indicates better model fit. The substantially lower AIC of the mixed logit confirms that individual-level preference heterogeneity is present. AIC = Akaike Information Criterion.
