# Supplementary material for: Prescriber’s Preferences for Digital Health Applications in Mental Health Care: Cross-Sectional Best-Worst Scaling Study of General Practitioners and Psychotherapists in Germany
Source: J Med Internet Res. 2026 Jul 8;28:e99203. doi: 10.2196/99203 (PMC13392533; doi:10.2196/99203)
Supplement: Multimedia Appendix 6 [file jmir_v28i1e99203_app6.doc]

Supplement 1: Mixed Logit Sensitivity Analysis– General Practitioners.

| Object | OR (CLogit) | Mean OR (MXL) | σ (MXL) | Direction consistent |
| --- | --- | --- | --- | --- |
| Alignment with scientific recommendations | 1.723 | 2.701 | 2.163 | Yes |
| Patient interest in using DiGA | 1.696 | 2.409 | 1.887 | Yes |
| Ability to tailor content to patient needs | 1.343 | 1.572 | 1.591 | Yes |
| Technical reliability | 1.056 | 1.111 | 0.600 | Yes |
| Contact point for technical support | 0.955 | 0.912 | 0.605 | Yes |
| Reimbursement of DiGA-related effort | 0.884 | 0.823 | 2.362 | Yes |
| Permanent listing in DiGA directory | 0.642 | 0.526 | 1.756 | Yes |
| Positive prior information/reputation | 0.630 | 0.448 | 1.370 | Yes |
| Availability on different devices | 0.568 | 0.313 | 2.572 | Yes |
| Continuous access to patient data | 0.467 | 0.276 | 2.614 | Yes |

Note: OR (CLogit) = odds ratio from stratified conditional logit (primary model, Error: Reference source not found). Mean OR (MXL) = exponentiated mean coefficient from mixed logit (μ). σ (MXL) = standard deviation parameter. Values substantially above 0 indicate individual-level heterogeneity. Direction consistent (Yes): CLogit and MXL mean OR lie on the same side of 1.0. Reference category: Intuitive usability for patients.
