# Supplementary material for: Prescriber’s Preferences for Digital Health Applications in Mental Health Care: Cross-Sectional Best-Worst Scaling Study of General Practitioners and Psychotherapists in Germany
Source: J Med Internet Res. 2026 Jul 8;28:e99203. doi: 10.2196/99203 (PMC13392533; doi:10.2196/99203)
Supplement: Multimedia Appendix 7 [file jmir_v28i1e99203_app7.doc]

Supplement 1: Mixed Logit Sensitivity Analysis – Psychotherapists.

| Object | OR (CLogit) | Mean OR (MXL) | σ (MXL) | Direction consistent |
| --- | --- | --- | --- | --- |
| Availability on different devices | 1.465 | 2.254 | 4.015 | Yes |
| Contact point for technical support | 1.361 | 1.857 | 1.144 | Yes |
| Positive prior information/reputation | 1.219 | 1.811 | 2.400 | Yes |
| Continuous access to patient data | 1.190 | 1.114 | 2.878 | Yes |
| Technical reliability | 1.158 | 1.359 | 0.148 | Yes |
| Permanent listing in DiGA directory | 1.117 | 1.411 | 1.776 | Yes |
| Patient interest in using DiGA | 1.098 | 1.077 | 1.912 | Yes |
| Ability to tailor content to patient needs | 1.087 | 1.220 | 1.476 | Yes |
| Reimbursement of DiGA-related effort | 0.999 | 1.141 | 1.739 | No |
| Alignment with scientific recommendations | 0.751 | 0.721 | 3.207 | Yes |

Note: OR (CLogit) = odds ratio from stratified conditional logit (primary model, Error: Reference source not found). Mean OR (MXL) = exponentiated mean coefficient from mixed logit (μ). σ (MXL) = standard deviation parameter. Values substantially above 0 indicate individual-level heterogeneity. Direction consistent (Yes): CLogit and MXL mean OR lie on the same side of 1.0. Reference category: Intuitive usability for patients.
