# Supplementary material for: Prescriber’s Preferences for Digital Health Applications in Mental Health Care: Cross-Sectional Best-Worst Scaling Study of General Practitioners and Psychotherapists in Germany
Source: J Med Internet Res. 2026 Jul 8;28:e99203. doi: 10.2196/99203 (PMC13392533; doi:10.2196/99203)
Supplement: Multimedia Appendix 8 [file jmir_v28i1e99203_app8.doc]

*Supplement 1: Sample representativeness.*

|  |  | ***GPs sample*** | ***GPs KBV*** | ***PSYs sample*** | ***PSYs KBV*** |
| --- | --- | --- | --- | --- | --- |
| ***Age (years) (%)*** | *under 40* | *11.7* | *8.0* | *24.8* | *18.4* |
|  | *40-50* | *23.4* | *24.4* | *30.2* | *28.2* |
|  | *50-60* | *36.3* | *30.4* | *21.8* | *23.0* |
|  | *over 60* | *28.9* | *37.1* | *23.3* | *30.4* |
| ***Sex (%)*** | *Female* | *50.7* | *51.3* | *75.2* | *77.7* |

*Note: PSY reference population based on Psychologische Psychotherapeuten (psychotherapists who did not study medicine but psychology), who comprised approximately 90% of the PSY sample. KBV data from gesundheitsdaten.kbv.de [6].*
